# Stroke Survivor’s Satisfaction and Experience with Rehabilitation Services: A Qualitative Systematic Review

**DOI:** 10.3390/jcm12165413

**Published:** 2023-08-20

**Authors:** Hanan Abu Saydah, Ruqayyah Turabi, Catherine Sackley, Fiona Moffatt

**Affiliations:** 1 Department of Medicine and Health Sciences, University of Nottingham, Nottingham NG7 2QL, UK; 2 Department of Physiotherapy, College of Applied Medical Sciences, Jizan University, Jizan 45142, Saudi Arabia; rturabi@jazanu.edu.sa; 3 School of Health Sciences, University of Nottingham, Nottingham NG7 2QL, UK; ntzfm@exmail.nottingham.ac.uk

**Keywords:** stroke, satisfaction, rehabilitation, experience, rehabilitative needs, rehabilitation service

## Abstract

Research in healthcare is increasingly focused on quality assurance and continuous quality improvement aiming to promote service quality. Satisfaction is a key endpoint in outcomes research and service benchmarking, along with “traditional” clinical outcomes. What controls stroke survivors’ satisfaction differs among qualitative studies’ conclusions, but there is general consensus on the importance of communication, improvement in activity, and engagement in goal setting. This review aims to collect and synthesise studies of the satisfaction of stroke survivors with rehabilitation services. A systematic search was conducted in seven electronic databases, including CINAHL, OVID, Pedro, Scopus Midline, Web of Science, and PubMed. The database search yielded 1339 studies, while one additional work was identified through hand searching. After removing duplicates, 74 studies were read in full, and after resultant exclusions, 12 qualitative studies were systematically reviewed, extracted, and appraised by two reviewers independently (HAS and RT) and the third reviewer (CS) was available for any disagreement. Five analytical themes were identified: Healthcare Professional–Patient Relationship (HCP), Delivery Service, Perceived Patient Autonomy (PPA), Expectations Shape Satisfaction, and Culture Influences Satisfaction. The studies of survivors’ satisfaction, experiences, and their rehabilitative needs with the services they receive have provided different factors that influence their satisfaction during rehabilitation in different countries worldwide. However, the context in which the studies were conducted is quite limited, and more detailed studies are required for many underexplored contexts.

## 1. Introduction

Survivors commonly report being dissatisfied with the rehabilitation services they receive in various contexts [1]. Factors or themes identified as controlling patient satisfaction differ among qualitative studies’ conclusions, but there is general consensus on the importance of communication, improvement in activity, and engagement in goal setting [2,3] Survivors’ dissatisfaction has been linked to “unmet needs” experienced by survivors as long-term care problems [4]. A wide range of long-term unmet needs among stroke patients has been documented in the literature, including with regard to physical activity, rehabilitation, and health-related care [5,6,7]. Following a preliminary search for a qualitative Systematic Review (SR) that focused on survivors’ satisfaction with rehabilitation, two reviews were found [8,9].

Peoples et al. [8] provided valuable knowledge on stroke experience during rehabilitation, but this study is relatively old, having been published over 10 years ago. Guidelines in healthcare have changed since that time, and different rehabilitation services have been introduced in terms of recent interventions, training, and telerehabilitation [10]. Consequently, it is crucial to gather more recent evidence on stroke satisfaction with rehabilitation. Luker et al. [9] synthesised a number of qualitative studies that explored survivors’ experiences with physical rehabilitation in an inpatient setting. Their review aimed to inform occupational therapists about different themes found to be related to survivors’ preferences in rehabilitation, but they focused only on inpatient experiences for only the occupational therapy component of rehabilitation; however, the majority of long-term rehabilitation services for survivors are now domiciliary (including the facilitation and support of patient self-care) [11]. Hence, due to the lack of a current SR that reflects the survivor’s satisfaction and experience with rehabilitation, this review aims to collect and synthesise studies of the satisfaction of survivors with rehabilitation services.

## 2. Materials and Methods

### 2.1. Inclusion and Exclusion Criteria

This qualitative SR will be reported and presented in a Preferred Reporting Items for Systematic Reviews and Meta-analyses extension for SR (PRISMA-ScR) flow diagram [12,13]. To start, a preliminary search for existing or ongoing SRs was conducted through the Cochrane Library, Joanna Briggs Institute (JBI), PROSPERO, and grey literature. The search strategy included unpublished and published research (restricted to English language). An initial limited search in MEDLINE, Cumulative Index of Nursing and Allied Health Literature (CINHAL), the Cochrane Database of Systematic Reviews, Cochrane Library, and the JBI Evidence Synthesis was undertaken to identify any possible keywords, synonyms, and Mesh terms related to this review topic. The text words contained in the titles and abstracts of relevant articles, and the index terms used to describe the articles, were used to develop a full search strategy in MEDLINE via Ovid, Embase, CINHAL, Pedro, and PubMed Sources for unpublished studies, and the grey literature to be searched were derived from Scopus, Web of Science, and Google Scholar.

The reference list of included studies was screened to identify additional studies. All databases were accessed via the Nottingham University (NUsearch) platform, and study authors were contacted via email by the researcher whenever there was missing information or any need for clarification. Identified citations were checked and uploaded into EndNote Insert X9/2018 (Clarivate Analytics, Philadelphia, PA, USA), and duplicates were removed (Table 1).

This protocol is now registered on PROSPERO with ID: CRD42022376532.

### 2.2. Assessment of Methodological Quality

Two reviewers (HAS and RT) appraised the studies, and they were blinded to each other’s assessments. This review used the standard JBI Checklist for Qualitative Research (JBICQR). Based on a comparative analysis of three online appraisal instruments’ ability to assess validity in qualitative research, the domains examined in the JBICQR were found to be more coherent and sensitive to the assessment of validity than other commonly used tools in qualitative studies [14]. Table 2 summarized the assessment of methodological quality of the included studies.

### 2.3. Data Extraction

The standardized data extraction tool from JBI SUMARI was used to extract the qualitative data from papers included in the review. This was completed by the primary reviewer (HAS) and was checked by the second reviewer (RT).

### 2.4. Data Synthesis

**Figure 1:**
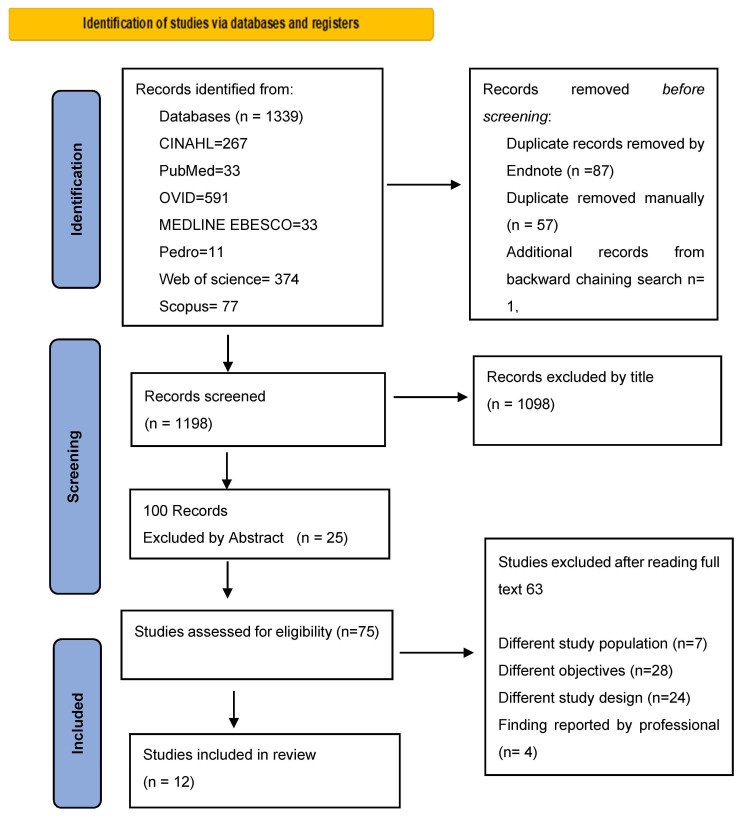
Identification of studies via databases and registers.

Qualitative research findings in this review underwent thematic synthesis to create new themes related to the SR question and objectives. This approach enables the author to produce new concepts and hypotheses while staying close to the primary finding from the studies [27]. Figure 1 displays the PRISMA diagram of the search process.

## 3. Results

### 3.1. Characteristics of Included Studies

#### 3.1.1. Time and Location

Studies were published in academic journals in the period 1995–2022. Although two of the included studies were published in the 1990s, and one was published in 2003, the remaining majority (*n* = 9) were published during the last 15 years. The studies represent a broad geographical distribution over nine countries, including one each for Canada [18], China [21] Denmark [20], Jordan [15], Nigeria [23], Norway [22] Sweden [28], and the UK [19]. However, a third of included studies (*n* = 4) were conducted in Australia [16,17,25,26].

#### 3.1.2. Methods and Methodologies

In-depth semi-structured interviews (SSIs) were conducted in all of the included studies to explore patients’ satisfaction, needs, and experiences with stroke rehabilitation, with the exception of two studies [18,23], which used focus group discussions with some participants following a survey.

The methodological approaches used in the studies varied. An ethnographic approach was used in two studies [15,21], whereas the majority used a phenomenological design [18,19,22,25,26]. Meanwhile, grounded theory was adopted in two studies [26,28], and a descriptive framework was adopted in one [17]. One of the studies did not state the methodological approach employed [20].

#### 3.1.3. Participants’ Demographic Characteristics

A total of 188 male and female survivors were included. One study had only four participants [23], while the maximum number was fifty [25]. The participants’ ages ranged from 29 to 85 years. Furthermore, the time following the stroke incident varied among the studies. Tomkins et al. [25] had the longest period post-stroke for aphasic stroke patients (4.5 years). A third of the studies used participants in the chronic stage (>six months) [17,25,26,28]. Al-Oraibi,S [15] studied participants 12–54 weeks after stroke [15], whereas two studies included stroke patients after three months [19,20]. Importantly, four included studies did not state the time post-stroke clearly [21,22,23,26].

#### 3.1.4. Phenomena of Interest

Satisfaction with general rehabilitation was explored as the phenomenon of interest in four included studies [15,22,23,25], while five studies focused on the experience of survivors with rehabilitation [18,19,20,26,28], and rehabilitative needs during rehabilitation for elderly and young survivors were captured in three studies [17,21,26]. Additionally, one of the included studies focused mainly on the experience of aphasic stroke patients and their needs following their stroke [25].

#### 3.1.5. Settings

This SR aimed to include studies in which survivors were able to reflect on their satisfaction, which entailed that they had already been discharged from hospital. However, two of the included studies did not mention the setting for the data collection process [17,23]. Interviews were arranged at a time and place that suited each participant in the other studies. In five included studies, the interviews took place in the participants’ homes [15,19,25,26,28], one of the studies used community association rehabilitation centre meeting rooms [18], and two used an out-patient department in addition to other settings [15,22]. One of the studies completed the interview either by telephone or via Skype, due to the national recruitment approach adopted, which prevented closer physical proximity and contact [26].

### 3.2. Summary of Findings

The relationship between the findings was examined and combined based on their shared characteristics. Five analytical themes were identified:

- HCP-Patient Relationship;
- Delivery Service;
- Perceived Patient Autonomy (PPA);
- Expectations Shape Satisfaction;
- Culture Influences Satisfaction.

#### 3.2.1. HCP–Patient Relationship

The theme of HCP–patient relationship emerged from descriptive themes extracted from half of the studies included in this SR. The attitudes of the staff significantly affected the survivors’ ability to maintain their individuality and dignity, including negative actions and remarks made by HCPs, such as the following:

“[HCPs] just took my cigarettes and lighter away from me” [21].

“You know you won’t walk again” [25].

Survivors expressed their satisfaction with their therapists during the rehabilitation process in different settings, including the outpatient department, physiotherapy, occupational therapy, and during their stay in the hospital:

“He tried to help me”.

“[Named occupational therapist] was very good. She helped me to move my fingers” [15].

“They were more of a support” [20].

On the other hand, a sense of disappointment and dissatisfaction was reported, such as a lack of therapist attention to patients’ needs:

“He was busy chatting with others about other issues”.

“She was more interested in people who had got motor neuron diseases

I think rather than … aphasia” [25].

Concerns were also expressed regarding the amount of rehabilitation input:

“If I could get more therapy I might improve” [15].

Time is needed to develop better relationships between HCPs and their patients during rehabilitation; however, survivors reported dissatisfaction with lack of HCP continuity during their rehabilitation and described this experience as “time lost”:

“To me, it is like it is [negatively] affecting the treatment” [20].

#### 3.2.2. Service Delivery

Participants expressed their satisfaction with the equipment available to them in the rehabilitation setting because it allowed for better training and good movement during training:

“Using equipment such as springs, pulleys… very useful” [18].

Conversely, younger stroke patients commonly reported a lack of flexible rehabilitation training, and those who were offered such services found that they did not match their needs and desires, which affected their experiences and thoughts about getting better in rehabilitation:

“Later, when I got home and faced reality, I realised that I needed more support” [24].

One participant was placed in a geriatric ward despite being only 52 years old. Consequently, the surroundings and activities that were offered did not match the participant’s needs, and they felt that they were “walking alongside” the process of rehabilitation [24]. Additionally, some of the survivors complained about having no suitable equipment in some community settings for their recovery, and they reported they could only receive basic medical help, such as blood pressure measurement:

“The clinic in our area has nothing for my disease, no exercises” [15].

Moreover, the privacy and crowding in other rehabilitation settings were reported as factors of dissatisfaction among survivors:

“So crowded with no privacy”.

“Waited a long time”.

“Prefer to have my therapy at home” [18].

In terms of follow-up, either in a rehabilitation setting or in participants’ homes, patients showed dissatisfaction with the lack of follow-up meetings with their regular therapist and

“Got annoyed with [having] no follow-up when I got back [home]” [22].

Looking at the studies conducted in remote areas, such as in Northern Australia, revealed a more acute lack of supportive care. For instance, Aboriginal people appeared to struggle to receive appropriate follow-up and transportation to attend rehabilitation appointments:

“It was just my wife at home, and my son”.

“A taxi was sent for me, but it wasn’t on a regular basis”.

“Family members provided support to patients” [17].

In Quebec, survivors reported a lack of assistance when getting home or follow-up care, so they had to engage family members during the recovery process, and they experienced difficulties finding an appointment for rehabilitation sessions:

“Difficulty obtaining assistance or care at home”.

“Had to be very persistent before receiving an outpatient appointment” [18].

These findings indicate the struggles that survivors experienced and the dissatisfaction they consequently experienced with the healthcare services they received.

#### 3.2.3. Perceived Patient Autonomy (PPA)

PPA is a term that refers to a person’s right to be involved in their care journey and to express their ability to participate [29]. In this context, patients’ perceptions of their own autonomy in their healthcare choices were related to whether HCPs “treat patients with humanity, and encouragement from therapists during training sessions strongly influenced satisfaction” [22].

Conversely, a sense of low PPA was commonly reported in relation to patients’ perceptions of how they were treated during their rehabilitation:

“It has something to do with being… We’re just sick people, nothing else” [19].

Elderly survivors stressed their special rehabilitative needs; they needed more information and education about their status (“I’m not well educated”) [21] to be involved in decision making about their therapy, and to be personally valued and acknowledged with respect. Moreover, they wanted to have their worries listened to; they wanted a caring attitude and to be respected as individuals within the scope of “what is available to me” [21]. These factors in PPA seem to be instrumental in patients’ satisfaction with rehabilitation in the stroke population generally, and particularly among the elderly.

#### 3.2.4. Expectations Shape Satisfaction

Satisfaction with rehabilitation appears to be met when certain factors relating to the survivor’s expectations from the services provided or from HCPs are achieved. Three of the included studies reported that participants were very optimistic regarding recovery following the stroke:

“With physiotherapy, we can have full recovery” [26].

More adherence and compliance with therapy inherently promote stroke recovery improvement. However, some participants did not expect much by the end of their therapy journey and held a grim view that it would be difficult to resume a “normal” life after the rehabilitation process. This was particularly acute among those who had initially experienced high levels of optimism, which they subsequently came to regard as unrealistic:

“There will be no treatment for a patient that can be adequate”.

“You wonder how you are going to cope”.

“I thought I’d just come out and be as good as gold” [28].

Importantly, patients who expected to return to complete normality (i.e., full recovery after rehabilitation with no long-term stroke impacts) were notably less satisfied with the services they received. Lawrence et al. [19] reported that a key issue in patients’ satisfaction is that pre-existing expectations of the patients about their HCPs may adversely affect their therapy, which was reflected in dissatisfaction reported during interviews:

“Care providers seemed to know little about stroke and its consequences, such as aphasia” [19].

The previously reported findings reflect how expectations may influence the sense of survivors’ satisfaction.

#### 3.2.5. Culture Influences Satisfaction

Two of the included studies highlighted the influence of cultural background on survivors’ satisfaction. Gender preferences in therapy sessions were also highlighted in one study conducted in the Middle East, because female patients expressed difficulties in being treated by males. If they needed home care therapy, they might ask for a non-HCP women to help because only male therapists are able to provide home care:

“Only male therapists work with survivors in their homes in Jordan” [15].

Religious or spiritual needs could be one factor contributing to patients’ satisfaction in rehabilitation. In China, for example, participants expressed their need to pray, which reflects the possible benefits of spiritual care for stroke patients, a hitherto underexplored area of stroke rehabilitation:

“Often pray to have peace and good health; it’s helpful to pray at that time and I feel better psychologically. Good, it’s helpful to pray” [21].

A key aspect found in this review was the collaboration between patients and HCPs. Thompson [30] suggested that the unique nature of the relationship between patients and professionals relates to both the public and private lives of individuals being emotionally and physically “exposed” in ways not conventionally expected. A “boundary-open” relationship with blurred boundaries responds to a patient wish to be “cared about” and not necessarily “cared for” [31], reflecting how expectations may develop during the treatment journey. Dissatisfaction was found to be linked to the level of attention HCPs gave during rehabilitation to make survivors relaxed and informed.

## 4. Discussion

### 4.1. Overview

It is essential to explore survivors’ satisfaction with rehabilitation services because of the importance of reflecting on the actual views of survivors and improving the level of care provided in practice. The relationship between the findings in this SR were identified in terms of five overarching themes: HCP–Patient Relationship, Delivery Service, Perceived Patient Autonomy (PPA), Expectations Shape Satisfaction, and Culture Influences Satisfaction. Survivors expressed their satisfaction with the help and support they received in different settings, such as physiotherapy [15,23] and occupational therapy [20].

In this review two studies reported that patients were dissatisfied either with the amount of rehabilitation provided or the type of training [20,21], which can inspire help-seeking in other rehabilitation settings. Lui et al. [21] reported that patients were disappointed about being forced to find private physiotherapy since their training terminated with an ongoing need for rehabilitation. Despite clinical guideline recommendations and updates, it seems that the amount of rehabilitation, intensity, and frequency needs remain unmet globally. A recent mixed-method study by Clark et al. [32] identified information exchange (one-to-one and in groups) as the pre-eminent determinant of the number of therapy sessions and their effectiveness. Although some therapists show an understanding of the evidence underpinning the recommendations for increasing therapy time, staff levels (i.e., low HCP-to-patient ratios) preclude their ability to deliver a sufficient duration of therapy for all patients.

### 4.2. Service Delivery

An enriched Service Delivery environment seems to draw positive impressions from survivors during rehabilitation, and increases their satisfaction and hopes of possible recovery [18]. As previously found in a published SR, the physical environment of rehabilitation is instrumental in survivors’ satisfaction, yet it has received limited attention in the literature [9]. Furthermore, there is a preference to have therapy at home, instead of attending the community-based rehabilitation and outpatient departments, which is attributed to the facilities available in local rehabilitation centres and their drawbacks, such as overcrowding, lack of privacy, and long waiting times. Follow-up meetings were key concerns identified by three studies in this review, as survivors in remote area expressed the struggle with care needed after discharge. Carlos et al. [33] found that patients with multiple chronic conditions receiving outpatient follow-up within 14 days after discharge was associated with a 1.5% reduction in readmissions in the lowest risk strata (*p* < 0.001). This indicates the importance of regular follow-up, not only to reduce readmission (which reduces long-term healthcare costs), but also to keep survivors motivated to recover and to stay satisfied.

PPA refers to patients’ right to express thoughts and choices during decisions related to their medical status [34]. In this SR, survivors reported their need for more respect and privacy in this regard [21].

Despite rehabilitation satisfaction studies tending not to consider the age of patients and time post-stroke, some researchers noted that elderly survivors had rehabilitative needs that differed from their younger counterparts, including with regard to autonomy, and information delivery (e.g., one participant was dissatisfied with medicalised information delivery, complaining that “I’m not well educated”) [21]. This suggests a need for flexible care, particularly in terms of delivering information (e.g., information on prognosis and self-care recommendations).

The convenience that survivors require in rehabilitation is not only linked to the training program, but also to being acknowledged as individuals and being involved when decisions are made [35]. However, support and information needs were noted as general issues among survivors of all ages, concurring with a pilot survey conducted with adult patients attending a Physiotherapy Department in Kuwait, where Sadeq et al. [36] indicated the importance of providing more attention, orientation, and information to older and less-educated stroke patients who may feel lost within a process they do not fully comprehend. A possible interpretation of the particular salience of informational needs among stroke patients is that their cognitive recall may be impaired due to the impacts of stroke itself and/or due to stress [37]. However, the intrinsic quality of information delivery by HCPs is also obviously instrumental. Some healthcare facilities may lack a systematic procedure of delivering information to patients (e.g., information about training, the time needed for each session, possible level of recovery, and availability of follow-up).

### 4.3. Expectations

Another finding was concerning how expectations can shape satisfaction; what survivors expect of their experience with rehabilitation, and the healthcare services provided in general must be considered by healthcare planners, policy makers, and managers because this experience, as much as the technical Quality of Care, will determine how people use the system and how they benefit from it [38]. In this review, expectations of rehabilitation were found to differ among survivors, but it seems that they generally hold strong beliefs in the ability of certain rehabilitation interventions to help them improve (particularly physiotherapy) [19,26,28].

Expectations about what healthcare services can provide to patients have been studied widely in different contexts, such as in emergencies, during which 70% of healthcare dissatisfaction was related to real or perceived problems involving physician communications, which influence patients’ expectations [39]. Not meeting expectations can also result in non-compliance or suboptimal compliance, and reduce the effectiveness of and satisfaction with medical care delivered by physicians in particular. In this review, the level of expectation varied between survivors, with some struggling with recovery (like coping with new situations) and others suffering in relation to physicians (care providers seeming to know little about stroke and its consequences, such as aphasia) [19,28]. Pertinent factors influencing expectations include survivors’ background, education, and stroke severity [40]. In this review, the chronicity level of stroke was over six months in more than half of the reviewed studies, which reflects the impacts of age and experience while reporting their satisfaction. Section 3.1.3 explains the socio-demographic characteristics of participants.

### 4.4. Cultural Differences

Cultural differences include factors related to beliefs, languages, behaviours, and prevailing national cultural characteristics [41]. The influence of culture on survivors’ satisfaction throughout the literature has been given limited attention. Wider literature, including a study considering the “influence of cultural background on motivation for and participation in rehabilitation following traumatic brain injury” revealed a higher level of distress among participants with minority culturally and linguistically diverse (CALD) backgrounds, and poorer outcomes in mobility and social integration when compared to other groups (i.e., the dominant English-speaking culture in Australia) [42]. In this review, gender preferences in rehabilitation were cited by one study in relation to the cultural background of participants in Jordan. Al-Oraibi, S. et al. [15] reported that female survivors always preferred female therapists, which profoundly affects rehabilitation services. This is related to an aversion to male–female physical contact among people not related by blood or marriage in Arab-Islamic cultures; although generally considered acceptable in the provision of biomedical care, it can be viewed with disapprobation for ancillary and supportive care, such as stroke rehabilitation (e.g., touching during physiotherapy). Religious factors also seem to influence survivors’ satisfaction, as expressed by participants in one included study who prayed to help with relief and recovery [21].

### 4.5. Study Limitations

Limitations of this review are related to the nature of the studies included and therefore include the factors discussed in the above analysis. For example, in terms of study design, a lack of theoretical framework, and an absence of details concerning data collection time and analysis were prevalent problems. Other limitations concerned the trustworthiness of data relate to the qualitative and subjective analysis of participants’ descriptions, for example, the rich presentation of participants’ responses, reliability checks, and detailed information on how the analysis were performed. Furthermore, the time post-stroke (i.e., the time since the patient had a stroke when data collection took place) was not mentioned in four studies, which raises questions regarding the accuracy of the reported satisfaction and experience with rehabilitation [21,22,23,26].

### 4.6. Methodological Weaknesses of the Review

Despite the generally good quality of the reviewed studies, some did not provide a clear definition of satisfaction with rehabilitation itself, given that it is a subjective experience among individuals. Further, a pragmatic decision was made to include only papers published in the English language, which led to the exclusion of four potentially relevant studies due to being published in other languages.

## 5. Conclusions

Studies of survivors’ satisfaction and their rehabilitative needs with the services they receive yielded different factors influencing their satisfaction during rehabilitation in different countries worldwide, but the context in which the studies were conducted were quite limited, and more detailed studies are required for many underexplored contexts.

## Figures and Tables

**Table 1:** Inclusion and exclusion criteria.

PICO Dimension	Criterion	
	Inclusion	Exclusion
Population	Studies exploring stroke survivor satisfaction, experience, and rehabilitative needs in rehabilitation, in rehabilitation settings including outpatient departments, community settings, private rehabilitation settings, and the home (in case of early supported discharge studies, contexts were included)	Studies exploring non-stroke populations, or stroke populations where findings on stroke population satisfaction cannot be extracted separately Stroke with cognitive deficit Memory loses
	Adult participants (aged 18 years old+)	Studies focused on the perspective of HCPs or family members
	Male and female	
	With and without aphasia	
Intervention (phenomena of interest)	Stroke survivors’ satisfaction with rehabilitation	
	Stroke survivors’ rehabilitation experiences and needs	
Context	Stroke survivor’s satisfaction with rehabilitation in out-patient departments, or in the community setting private rehabilitation settings	Qualitative studies that focused only on inpatient experiences
	Studies of stroke satisfaction with rehabilitation in early supported discharge	
	Stroke survivors’ rehabilitation experiences in nursing homes	
Outcome (type of study)	Primary empirical qualitative study designs, including but not limited to; phenomenology, grounded theory, ethnography, action research, and feminist research	Case report studies, commentary papers, and quantitative studies (including randomised controlled trials, quasi-experimental studies, cohort studies, and reviews)
	Mixed studies designs that explored the topic area, and use the qualitative part in informing the research question	Qualitative studies not reporting participant experiences (participants’ voices).
	Published in peer-reviewed journals	
	Written in the English language	
	No restrictions for geographical locations or time of publication	

**Table 2:** Assessment of methodological quality.

Study Criterion	Al-Oraibi, S. 2011 [15]	Hoffmann, 2020 [16]	Kelly, 2022 [17]	Lamontagne, 2018 [18]	Lawrence et al., 2012 [19]	Lewinter et al., 1995 [20]	Lui et al., 1999 [21]	Mangset et al., 2008 [22]	Olaleye, 2017 [23]	ROiDING et al., 2003 [24]	Tomkins et al., 2013 [25]	White et al., 2009 [26]
1. Is there congruity between the stated philosophical perspective and the research methodology?	Yes	Yes	Yes	Yes	Yes	No	Yes	Yes	Yes	No	Yes	Yes
2. Is there congruity between the research methodology and the research question or objectives?	Yes	Yes	Yes	Yes	Yes	Yes	Yes	Yes	Yes	Yes	Yes	Yes
3. Is there congruity between the research methodology and the methods used to collect data?	Yes	Yes	Yes	Yes	Yes	Yes	Yes	Yes	Yes	Yes	Yes	No
4. Is there congruity between the research methodology and the representation and analysis of data	Yes	Yes	Yes	Yes	Yes	Yes	Yes	Yes	Yes	Yes	Yes	Yes
5. Is there congruity between the research methodology and the interpretation of results?	Yes	Yes	Yes	No	Yes	Yes	Yes	Yes	Yes	Yes	Yes	Yes
6. Is there a statement locating the researcher culturally or theoretically?	No	Yes	Yes	No	No	No	No	No	No	No	No	Yes
7. Is the influence of the researcher on the research, and vice- versa, addressed?	No	Yes	Yes	No	No	No	No	Yes	Yes	No	No	No
8. Are participants, and their voices, adequately represented?	Yes	Yes	Yes	No	Yes	No	Yes	Yes	Yes	Yes	Yes	Yes
9. Is the research ethical according to current criteria or, for recent studies, and is there evidence of ethical approval by an appropriate body?	Yes	Yes	Yes	No	Yes	No	Yes	Yes	No	Yes	No	Yes
10. Do the conclusions drawn in the research report flow from the analysis, or interpretation, of the data?	Yes	Yes	Yes	Yes	Yes	Yes	Yes	Yes	Yes	Yes	Yes	Yes
Total score	8/10	10/10	10/10	5/10	8/10	5/10	8/10	9/10	8/10	7/10	7/10	8/10
The decision of inclusion	Yes	Yes	Yes	Yes	Yes	Yes	Yes	Yes	Yes	Yes	Yes	Yes

## Data Availability

Not applicable.
